# 1396. The Incremental Hospitalization Burden Associated with Nontuberculous Mycobacterial Lung Disease(NTMLD) among Patients with Chronic Obstructive Pulmonary Disease (COPD) in Japan

**DOI:** 10.1093/ofid/ofab466.1588

**Published:** 2021-12-04

**Authors:** Naoki Hasegawa, Kozo Morimoto, Ping Wang, Lu Zhang, Mariam Hassan, Anjan Chatterjee

**Affiliations:** 1 Keio University Hospital, Tokyo, Tokyo, Japan; 2 Fukujuji Hospital, Tokyo, Tokyo, Japan; 3 Insmed Incorporated, Bridgewater, New Jersey; 4 Panalgo, Boston, Massachusetts

## Abstract

**Background:**

NTMLD is a life-threatening pulmonary infection with increasing incidence and prevalence in Japan. It is associated with progressive lung damage and increased healthcare use. Many patients with NTMLD have comorbid respiratory conditions such as COPD. Treatment of NTMLD in patients with COPD is difficult, however there is limited data on the incremental burden that NTMLD adds to underlying COPD. We assessed the incremental burden associated with NTMLD in Japanese patients with COPD by comparing their hospitalizations to matched COPD patients without NTMLD.

**Methods:**

A retrospective cohort study was conducted using claims data provided by the Japan Medical Data Center (2015-2020). COPD patients with NTMLD were matched 1:3 to COPD patients without NTMLD (controls). Hospitalizations (all-cause, respiratory-related, and COPD-related) were accrued over a 1-year follow-up period after NTMLD diagnosis (index). Incremental burden of NTMLD was assessed by comparing hospitalizations between COPD patients with NTMLD and controls with univariate and multivariate analyses adjusting for comorbidities during 1-year pre-index period.

**Results:**

A total of 492 COPD patients with NTMLD were matched by age and sex to 1476 controls. Mean (SD) age on index date was 56.6 (10.3) years and 61.4% were females. Compared to controls, NTMLD patients had higher prevalence of some pulmonary symptoms and comorbidities such as hemoptysis (11% vs 2%), dyspnea (1.6% vs 0.6%) and lung cancer (7% vs 4%). In univariate analyses, a higher percent of COPD patients with NTMLD had hospitalizations compared to controls (Fig 1A); the unadjusted annual hospitalization rates were also higher among patients with NTMLD (Fig 2A). Multivariate regressions after adjusting for pre-index comorbidities showed COPD patients with NTMLD were 1.9 times more likely to have an all-cause hospitalization, 2.8 times more likely to have a respiratory hospitalization, and 3.0 times more likely to have a COPD-related hospitalization (Fig 1B).

**Conclusion:**

COPD patients with NTMLD had a higher burden of hospitalization than COPD patients without NTMLD. The statistically significantly incremental burden associated with NTMLD in patients with COPD highlights the acute need for appropriate management of NTMLD in Japan.

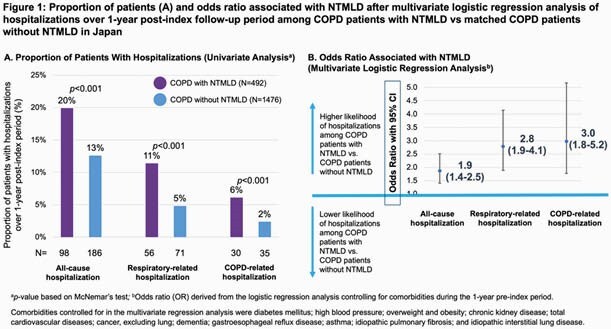

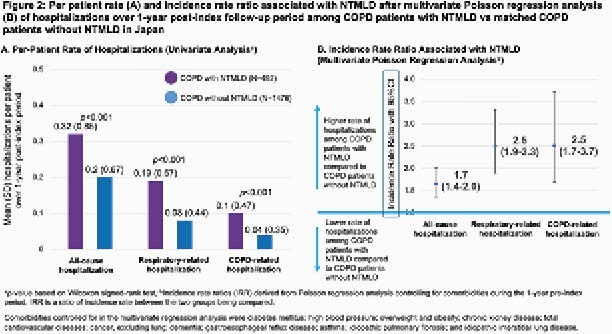

**Disclosures:**

**Naoki Hasegawa, MD, PhD**, **Insmed Incorporated** (Consultant, Scientific Research Study Investigator)**Janssen Pharmaceuticals Inc** (Consultant, Scientific Research Study Investigator) **Kozo Morimoto, MD**, **Insmed Incorporated** (Consultant) **Ping Wang, PhD**, **Insmed Incorporated** (Employee) **Lu Zhang, PhD**, **Panalgo** (Employee, Other Financial or Material Support, Lu Zhang is an employee of Panalgo which provides the analytic platform Instant Health Data that is used by Insmed) **Mariam Hassan, PhD, B. Pharm**, **Insmed Incorporated** (Employee) **Anjan Chatterjee, MD, MPH**, **Insmed Incorporated** (Employee)

